# Teen Dating Violence Victimization: Associations Among Peer Justification, Attitudes Toward Gender Inequality, Sexual Activity, and Peer Victimization

**DOI:** 10.1177/08862605221085015

**Published:** 2022-04-12

**Authors:** Kristen E. Hunt, Luz E. Robinson, Alberto Valido, Dorothy L. Espelage, Jun Sung Hong

**Affiliations:** 1 School of Education, 2331 University of North Carolina, Chapel Hill, USA; 2 School of Social Work, 2954 Wayne State University, Detroit, MI, USA

**Keywords:** youth violence, bullying, dating violence, domestic violence, victimization

## Abstract

The current study, grounded in a social learning theoretical framework, examined attitudes and behaviors associated with verbal and physical teen dating violence (TDV) victimization. Because TDV varies by gender in both frequency and severity, these associations were examined first within the overall sample, and then by gender to further investigate these differences. A total of 1,884 adolescents (49.2% boys; 50.8% girls; average age 14.79 years; *SD* = .58) who reported ever dating someone were included in the analysis. Specifically, peers’ justification of TDV, attitudes supporting gender inequality, sexual activity, and peer victimization were included to determine their cross-sectional association with verbal and physical TDV victimization. Data were analyzed separately for boys and girls. Results indicated that peers’ justification of TDV, peer victimization, sexual activity, and attitudes supporting gender inequality were each associated with higher physical and verbal TDV victimization for girls and boys. Most of these factors remained significant when separated by gender, except for sexual activity and attitudes supporting gender inequality, which were not associated with physical TDV victimization for boys and girls, respectively. Implications for practice and research are discussed.

Teen dating violence (TDV) is a serious public health concern affecting children and adolescents across the United States (U.S.). The Centers for Disease Control and Prevention defines TDV as a type of intimate partner violence between two adolescents that includes physical violence, sexual violence, psychological aggression, and/or stalking (Centers for Disease Control and Prevention, 2020). In the U.S., approximately 400,000 adolescents are victims of TDV (Wolitzky-Taylor et al., 2008). TDV has many negative consequences for its victims, including depression, substance abuse, and unhealthy eating behaviors (Centers for Disease Control and Prevention, 2020; Exner-Cortens et al., 2013; Kannegieter, n.d.). To effectively counter the cycle of TDV violence, researchers need to identify the most consistent risk factors and use these data to prevent its occurrence.

The current study aims to better understand the risk factors associated with TDV victimization, as identified by extant literature, to inform prevention efforts that may be different for adolescent females and males. Research generally indicates gender differences in TDV, although they are not always consistent. Generally, girls are more frequently victims of TDV (Ackard et al., 2003), whereas boys are more likely to inflict physical violence in dating relationships (Sears et al., 2006). Research has also documented that girls perpetrate more verbal aggression, while they are victimized by psychological and sexual aggression more often than boys (Sears et al., 2006). However, empirical findings regarding gender differences have been inconsistent, likely because of variability in the measurement of TDV and sampling methodologies (Wincentak et al., 2017). For example, some studies have reported that both boys and girls are equally likely to be victims of verbal and physical TDV (Lewis & Fremouw, 2001; Wincentak et al., 2017). However, another study documented differences with about one-third of girls (31.5%) and more than one-fourth of boys (26.4%) reporting physically aggressive behaviors toward their intimate partners (Simon et al., 2010). Thus, the need to examine existing risk factors and their association with verbal and physical TDV victimization for female and male adolescents is imperative to informing TDV prevention initiatives.

## Theoretical Framework

While many existing studies investigate TDV victimization through a feminist lens or family context (Garthe et al., 2019; Ustunel, 2020), the current study examined each factor within a social learning theoretical framework to emphasize the importance of social norms, interpersonal conflicts (i.e., peer victimization), and the social network of peers during adolescence. According to social learning theory, proposed by Albert Bandura, people learn how to behave by imitating the behaviors they are exposed to in their environment (Bandura & McClelland, 1977). Similarly, this theory relies heavily upon observational learning, a process by which people observe the behaviors of those around them, retain the information, and later replicate or mimic the behaviors themselves. Behaviors that are mimicked are often reinforced or expected to be reinforced when perceived to align with social norms. A key understanding of observational learning is that humans are more likely to attend to and imitate behavior from people who they perceive as being similar to themselves (Bandura, 2008).

Social learning theory helps to explain not only the consequences of behavior, but also the cognitive processes that precede and shape behaviors (Bandura, 2008). Applying social learning theory, the peers’ approval of violent behaviors (whether through verbal or behavioral indications) would likely shape an individual’s cognitive processes and behaviors to justify these abusive behaviors for themselves. Similarly, attitudes regarding gender inequality, which delineate differential expectations and distributions of power, may be introduced in the family context, and are also shaped by observational learning among peers during adolescence (Kågesten et al., 2016). Examples could include the expectation that boys in relationships should pay for everything on a date or the difference in social stigma between a promiscuous boy and a promiscuous girl. Relatedly, sexual behavior during adolescence is also influenced by the peer context, which often reinforces gender stereotypes (Metzler et al., 1994). Finally, numerous studies have demonstrated that interpersonal victimization in one context is often associated with other types of victimization in other contexts (Espelage et al., 2012; Hong et al., 2016). Therefore, adolescents who experience peer victimization may also experience victimization in their romantic relationships.

Each of these predictors was examined separately for boys and girls, in accordance with social learning theory. Because humans are most likely to imitate the behaviors of individuals they perceive similar to them (Bandura, 2008), and because boys and girls are socialized from an early age to spend most of their time with peers of the same gender (Cohen, 2001), we can expect that boys and girls will experience TDV physical and verbal victimization differently.

## Peer Justification of TDV

The literature on dating violence consistently shows a positive relationship between risky peer networks and TDV (Chase et al., 2002). Adolescents are particularly vulnerable to dating violence, primarily because during this stage of development many attitudes, beliefs, and perceptions around healthy intimate relationships are not yet formed. Adolescents spend most of their day at school, and their attitudes around dating are heavily influenced by what their peers are doing and who their peers are dating (Oudekerk et al., 2014). Research has shown that adolescents whose peer networks consist of individuals who are in abusive relationships or condone violent behaviors within relationships are more likely to adopt these attitudes or behaviors themselves (Capaldi et al., 2012; McDonell et al., 2010). For instance, McDonell et al.’s (2010) study of young people in the rural South found that students’ positive attitudes toward violence were positively related to dating violence victimization for both males and females.

As expected, attitudes shaped by peer influence have consistently been associated with TDV victimization compared to witnessing violence or abuse between parents at home (Arriaga & Foshee, 2004). For instance, one study that examined 223 youths with four categories of risk factors for dating violence—including risky social environment (peer influence), risky sexual history, risky family background, and poor ability to self-regulate—found that a risky social environment was the single most important factor in determining TDV victimization (Oudekerk et al., 2014). In other words, peer networks and exposure to peer dating violence are among the most important risk factors to consider for TDV prevention. Thus, this study examines peers’ justification of TDV.

## Attitudes Endorsing Traditional Gender Roles and Gender Inequality

Attitudes in support of gender inequality within society and relationships can foster TDV victimization. Research indicates holding attitudes that promote traditional gender roles that perpetuate gender inequality and promote violence against women (McCarthy et al., 2018) increases males’ odds of perpetrating dating violence (McCauley et al., 2013; Reed et al., 2011; Reyes et al., 2016; Shen et al., 2012; Tharp et al., 2011) and females’ risk of TDV victimization (Taquette & Monteiro, 2019). Reyes et al.’s (2016) study found that traditional gender role attitudes at Time 1 were positively associated with an increased risk for dating violence perpetration eighteen months later (Time 2). In a study of TDV among Chinese adolescents, Shen et al. (2012) also found that boys’ endorsement of traditional gender roles and boys’ attitudes justifying boy-to-girl dating violence were the strongest predictors of perpetrating physical and sexual dating violence. Taquette and Monteiro’s (2019) review of studies further indicated that TDV is deep-seated in the patriarchal culture and is linked to racism, heterosexism, and poverty. Although much of the literature examines perpetration as it relates to gender inequality, it is unclear how attitudes toward traditional gender roles and gender inequality are associated with TDV victimization among boys and girls.

## Sexual Behavior

While many factors must be considered when determining whether sexual behavior is risky or not, many studies have indicated that sexual behavior during adolescence should be considered risky in itself. More specifically, although relatively limited, extant studies report that sexual initiation before the age of sixteen is correlated with higher levels of TDV victimization (Lichter & McCloskey, 2004; Rodgers & McGuire, 2012). In addition to consequences such as teen pregnancy and the spread of sexually transmitted diseases, there are numerous emotional consequences linked to early sexual activity that likely affect vulnerability to TDV, such as altered self-esteem, depression, and greater difficulty forming healthy relationships (Malhotra, 2008). Lowered self-esteem and the reduced ability to form healthy relationships may explain the association between early sexual behavior and higher levels of TDV victimization. Furthermore, many of these consequences generally affect boys and girls differently (Metzler et al., 1994), and examining this predictor for boys and girls separately is therefore necessary for a more complete understanding of gender differences in this regard.

## Peer Victimization

Peer victimization is the experience of being a target of the aggressive behavior of other children, which can be verbal, physical, and/or relational aggression that tends to be repetitive, intentional, and associated with a power imbalance (Espelage & Holt, 2001; Hawker & Boulton, 2000). Both cross-sectional and longitudinal studies indicate that peer victimization is associated with adverse mental health outcomes for boys and girls, including depression, suicidal ideation, and low self-esteem (Polanin et al., 2021; Robinson et al., 2021; Wu et al., 2021). Peer victimization peaks during early adolescence (Centers for Disease Control and Prevention, 2019). As adolescents develop their own identities and gain more experience in interpersonal relationships with their peers, peer victimization impedes the development of future healthy interpersonal relationships. Incidents of peer victimization may normalize aggression in interpersonal relationships, including dating relationships (Cava et al., 2018).

Researchers have recognized the importance of investigating polyvictimization, how youths’ victimization in one context (e.g., victimization within the peer context) might escalate into victimization in another context (e.g., victimization in dating relationships). Existing longitudinal studies have shown that adolescents who are victimized by their peers are vulnerable to becoming victims of TDV (Brooks-Russell et al., 2013; Cava et al., 2018; Hipwell et al., 2014; Sabina et al., 2016). According to Cava et al.’s (2018) study, a positive association between peer victimization and dating violence victimization was found in a sample of 1,038 Spanish early and middle adolescents in a dating relationship. The association between peer victimization and TDV victimization has also been documented in cross-sectional research on co-occurring victimizations, which suggests that being victimized by peers is concurrently associated with being a victim of TDV in adolescence (Miller et al., 2013). However, it is important to examine whether peer victimization is associated with both physical and verbal TDV and whether these associations differ for boys and girls.

## Current Study

Utilizing a cross-sectional design, this study aims to identify whether the following attitudes and behaviors among high school students are associated with TDV victimization: (a) peers’ justification of TDV, (b) attitudes supporting gender inequality, (c) sexual activity, and (d) peer victimization. Other factors include age, gender, and race/ethnicity, as these may be associated with TDV victimization. Two types of TDV measured in the current study are verbal victimization and physical victimization as they are the most prevalent forms of TDV. Additionally, verbal victimization typically precedes or co-occurs with physical victimization, indicating different levels of severity. These findings will be used to inform the development of educational and preventive programs to identify attitudes and behaviors that, when addressed, might mitigate the risk of TDV victimization.

The hypotheses are as follows: H1: Higher level of peers’ justification of TDV will be associated with higher levels of verbal and physical TDV victimization; H2: Greater attitudes supporting gender inequality will be associated with higher levels of verbal and physical TDV victimization; H3: Sexual activity such as having had sexual intercourse will be associated with higher levels of verbal and physical TDV victimization; and H4: Experiencing peer victimization will be associated with experiencing verbal and physical TDV victimization. We did not have specific hypotheses for the differences in these associations between boys and girls. The current study utilized a structural equation modeling analysis separately for boys and girls.

## Methods

### Participants

Participants included 1,884 students who reported ever dating someone from six Midwestern U.S. high schools. Data were collected in October, 2014 and surveys were completed online. Participants completed a survey with 67 questions regarding family history, peer victimization, and dating violence in a single session. Of the 1,884 participants included in the sample, 927 boys and 957 girls completed the survey. In the final sample, the ages ranged from 13 to 17 years (*M* = 14.79; *SD* = 0.58). Participant demographics were as follows: 32% of participants identified as Black/African American, 30% identified as White, 23% identified as Hispanic, 1% identified as Asian, 3% identified as biracial, and 11% identified as multiracial.

### Procedure

A waiver of active parental consent was approved by the University of Illinois Institutional Review Board. A waiver of consent was approved for several reasons: (1) The purpose of the school-wide surveys is to assess students’ engagement in peer and dating violence perpetration and victimization and other predictors of TDV. Research assessing such self-reported attitudes and behaviors poses no more than minimal risk to participants, and to provide the best estimates of these factors, it was important to maximize the size and representativeness of the samples; (2) Previous research has documented not only lower participation rates in general when “active” consent procedures were employed (i.e., when written parental consent was required) but also reported important demographic and behavioral differences between samples obtained with active versus “implied” consent procedures (i.e., when parental non-response was taken to mean they had no objection to their child’s participation; Liu et al. 2017). For example, Liu et al. (2017) conducted a meta-analysis on peer-reviewed articles and unpublished dissertations from 1975 to 2016. Results showed that (1) the response rates were significantly lower for studies using active consent procedure than those using passive consent procedure; (2) more females and younger participants, and fewer Black/African American participants were included in studies using active consent procedures than studies using passive procedures; and (3) studies with passive consent procedures revealed higher rates of self-reported risk behaviors such as substance use than studies with active consent procedures.

The research team worked closely with each school to make certain that parents were aware of the survey through various outlets and made sure they had many options to opt their child out of the survey. Outreach to parents included presentations at parent-teacher association meetings, newsletter pieces, several email blasts to students, and parent informational letters via email and postal mail. Parents could opt out their child by calling or emailing the school or PI, returning the signed information form, or calling a teacher. Parents signed and returned an informational letter only if they did not want their child to participate in the study. Before starting the survey, trained proctors read an assent script to students, and students could elect not to participate and/or skip any questions. On average, students took 30 minutes to complete the survey and did so during regular school hours. The self-report survey was completed in a single session and data were de-identified immediately following data collection. Participants responded to a series of questions that inquired about peer victimization and dating violence. After the survey was completed, they were debriefed with the purpose of this study and given information about the services available at their school and in their community to address TDV and mental health concerns.

### Measures

#### Demographic Questionnaire

The demographic questionnaire measured age (in years), gender (female, male, transgender, or other), grade (9–12), and race/ethnicity (Black/African American, White, Hispanic, Asian/Pacific Islander, biracial, or Other race/ethnicity). Participants were allowed to identify with more than one race and those who did were recoded as multiracial. Because all participants who responded to gender listed “girl” or “boy,” only these genders were included in the analyses. Having dated was asked with one question, “Have you ever dated someone?” with response options, 0 (*No*) and 1 (*Yes*).

#### Verbal and Physical TDV Victimization

Verbal and physical TDV victimization were assessed with the Conflict in Adolescent Dating Relationships Inventory (CADRI), a measure of abusive behavior among adolescent dating partners (Wolfe et al., 2001). The verbal and physical TDV victimization scales were used in this analysis. To ensure participants understood dating, the following definition was provided: Spending time with someone you are seeing or going out with (one-time date, long-term relationship). Participants were asked how often in their lifetime they had experienced verbal (9 items) and physical (8 items) forms of TDV from a dating partner/s. Response options included 0 (*Never*), 1 (*Seldom, 1-2 times*), 2 (*Sometimes, 3-5 times*), and 3 (*Often, 6+ times*) on a 4-point Likert-type scale. In this study, each set of questions attempted to identify different types of abusive behavior, including verbal abuse and physical abuse. Examples of questions include, “He/she insulted me with put-downs” and “He/she pushed, shoved, or shook me.” Cronbach alpha coefficients were 0.88 for the verbal and 0.89 for the physical subscales in this study.

#### Attitudes Supporting Gender Inequality

Attitudes supporting gender inequality were assessed with an adapted measure of gender violence and harassment (Taylor et al., 2008). Participants were asked to indicate how strongly they agreed with statements that promoted gender inequality, such as, “In a dating relationship the boy should be smarter than the girl.” This measure consisted of 7 items. Response options included 0 (*Strongly Disagree*), 1 (*Disagree Somewhat*), 2 (*Agree Somewhat*), and 3 (*Strongly Agree*) on a 4-point Likert-type scale. Cronbach’s alpha coefficient was 0.77 for this study.

#### Peers’ Justification of TDV

Peers’ justification of TDV was assessed based upon responses to an adapted measure of peer attitudes toward TDV behaviors. Participants were asked to indicate how strongly they believed their friends agreed with various statements justifying TDV, such as, “My friends generally think that it is ok for a boy to hit his/her girlfriend if she did something to make him/her mad.” This measure consisted of one scale with eight items. Response options included 0 (*Strongly Disagree*), 1 (*Disagree Somewhat*), 2 (*Agree Somewhat*), and 3 (*Strongly Agree*) on a 4-point Likert-type scale. Cronbach’s alpha coefficient was 0.89 for this study.

#### Sexual Activity

Sexual activity was assessed with one item: “Have you ever had oral sex, anal intercourse, or vaginal intercourse (sometimes called making love, having sex, going all the way, getting laid, or screwing)?” Response options included 0 (*No*) or 1 (*Yes*).

#### Peer Victimization

The four-item University of Illinois Victimization Scale (Espelage & Holt, 2001) assessed victimization from peers. Participants were asked how often the following had happened to them in the past 30 days: “Other students called me names”; “Other students made fun of me”; “Other students picked on me”; and “I got hit and pushed by other students.” Response options included 0 (*Never*), 1 (*1–2 times*), 2 (*3–4 times*), 3 (*5–6 times*) and 4 (*7 or more times*) on a 5-point Likert-type scale. The construct validity of this scale has been supported by exploratory and confirmatory factor analysis (Espelage & Holt, 2001). Scores have converged with peer nominations of victimization (Espelage & Holt, 2001). Higher scores indicate more self-reported victimization. Cronbach's alpha coefficient was 0.78 for this study.

### Data Analysis

Descriptive statistics were computed for each dependent and independent variable separately for males and females and the overall sample.

Several path analysis models were computed using the SEM R package lavaan (Rosseel, 2012). Full information maximum likelihood (FIML) was used to handle missing data (Enders & Bandalos, 2001). The first set of models included the outcomes verbal and physical TDV victimization for the full sample. Both of these models included the predictors peers’ justification of TDV, peer victimization, sexual activity, attitudes supporting gender inequality, age, gender, and race/ethnicity (Black/African American, Hispanic, and other race/ethnicity; reference White). Given the documented differences between girls and boys on verbal and physical TDV victimization rates, we then conducted separate SEM analyses with groups for boys and girls with the same predictors and outcomes (excluding gender). As a sensitivity analysis, we also tested a series of moderation analyses to determine whether gender moderated the associations between peers’ justification of TDV, peer victimization, sexual activity, and attitudes supporting gender inequality on verbal and physical TDV victimization. However, none of the interactions with gender were significant and results are not presented for parsimony.

## Results

### Descriptive Statistics

Table 1 presents descriptive statistics for boys and girls and the overall sample. Independent sample *t*-tests suggest that on average, girls experienced significantly higher verbal TDV victimization when compared to boys. However, both girls and boys experienced similar levels of physical TDV victimization. Additionally, boys reported significantly higher levels of attitudes supporting gender inequality when compared to girls. Lastly, on average, girls reported significantly higher level of peer victimization when compared to boys.

**Table 1.** Descriptive Statistics.

		Overall (*N* = 1884)		Boys (*N* = 927)		Girls (*N* = 957)		Group Comparison
	Range	*M*/N	*SD*/(%)^a^	*M*/N	*SD*/(%)^a^	*M*/N	*SD*/(%)^a^	Hedge’s g
*Demographics*							
Age (in years)	13–17	14.79	0.58	14.82^b^	0.62	14.76	0.54	−0.10
13 years		2	(0%)	1	(0%)	1	(0%)	
14 years		538	(29%)	267	(29%)	271	(28%)	
15 years		1209	(64%)	614	(66%)	595	(62%)	
16 years		118	(6%)	40	(4%)	78	(8%)	
17 years		14	(1%)	4	(0%)	10	(1%)	
Race/ethnicity							
Black/African American		586	(32%)	284	(30%)	302	(33%)	
Hispanic		432	(23%)	230	(25%)	202	(22%)	
White		548	(30%)	287	(31%)	261	(29%)	
Other race/ethnicity		276	(15%)	133	(14%)	143	(16%)	
Asian		21	(1%)	14	(1.5%)	7	(0.8%)	
Biracial		55	(3%)	27	(2.9%)	28	(3.1%)	
Multiracial		200	(11%)	92	(9.9%)	108	(12%)	
*Predictors*							
Peers' justification of TDV	1–4	1.27	0.45	1.27	0.47	1.26	0.43	−0.02
Peer victimization	0–4	0.44	0.74	0.37	0.68	0.51^c^	0.80	0.20
Sexual activity (1 = yes)	0–1	637	(54%)	322	(56%)	315	(52%)	-
Attitudes supporting gender inequality	1–4	1.79	0.59	1.86^b^	0.64	1.72	0.53	−0.25
Verbal TDV victimization	0–3	0.44	0.54	0.37	0.50	0.52^c^	0.57	0.28
Physical TDV victimization	0–3	0.11	0.32	0.11	0.30	0.12	0.33	0.02

*Note.* TDV = teen dating violence. *M* = mean. *SD* = standard deviation.

^a^Percentages may not add to 100% due to rounding.

^b^Higher average than girls, independent sample t-test: *p*<.05.

^c^Higher average than boys, independent sample t-test: *p*<.05.

### Verbal TDV Victimization

Table 2 presents the results for the verbal TDV victimization model for the overall sample. Peers' justification of TDV was positively associated with verbal TDV victimization (*Est.* = 0.07, *SE* = 0.02, *p* < .001). That is, higher justification of TDV among peers was associated with higher verbal victimization. Similarly, higher levels of peer victimization were associated with higher verbal TDV victimization (*Est.* = 0.19, *SE* = 0.02, *p* < .001). Sexual activity (*Est.* = 0.18, *SE* = 0.03, *p* < .001) and attitudes supporting gender inequality (*Est.* = 0.11, *SE* = 0.02, *p* < .001) were also associated with higher verbal TDV victimization. Additionally, higher age (*Est.* = 0.02, *SE* = 0.01, *p* < .05) was associated with higher reports of verbal TDV victimization. Girls reported higher verbal TDV victimization when compared to boys (*Est.* = 0.15, *SE* = 0.02, *p* < .001). Lastly, Black/African American (*Est.* = −0.07, *SE* = 0.03, *p* < .05) and Hispanic students (*Est.* = −0.07, *SE* = 0.03, *p* < 0.05) reported lower verbal TDV victimization when compared to White students.

**Table 2.** Verbal and Physical Teen Dating Violence Victimization for the Entire Sample .

	Physical TDV		Verbal TDV	
	Est.	*SE*	Est.	*SE*
Peers' justification of TDV	0.05***	**0.01**	0.07***	**0.02**
Peer victimization	0.09***	**0.02**	0.19***	**0.02**
Sexual activity	0.05**	**0.02**	0.18***	**0.03**
Attitudes supporting gender inequality	0.04***	**0.01**	0.11***	**0.02**
Age	0.01	0.01	0.02*	**0.01**
Girls	−0.00	0.01	0.15***	**0.02**
Black/African American	0.01	0.02	−0.07*	**0.03**
Hispanic	0.03	0.02	-0.07*	**0.03**
Other race/ethnicity	0.06*	**0.03**	0.044	0.04

*Note.* TDV = teen dating violence. *SE* = standard error.

*** *p* < .001; ** p < .01; * p < .05; White was the reference category for race/ethnicity. Boys was the reference category for gender.

### Physical TDV Victimization

Table 2 presents results for the physical TDV victimization model for the overall sample. The model suggests that higher levels of peers' justification of TDV (*Est.* = 0.05, *SE* = 0.01, *p* < .001), peer victimization (*Est.* = 0.09, *SE* = 0.02, *p* < .001), sexual activity (*Est.* = 0.05, *SE* = 0.02, *p* < .01), and attitudes supporting gender inequality (*Est.* = 0.04, *SE* = 0.01, *p* < .001) were associated with higher reports of physical TDV victimization. Further, identifying as other race/ethnicity was associated with higher physical TDV victimization (*Est.* = 0.06, *SE* = 0.03 *p* < .05) when compared to White students.

### Verbal TDV Victimization by Gender

Table 3 presents results for the verbal TDV victimization models for boys and girls. In the model for girls, peers’ justification of TDV (*Est.* = 0.10, *SE* = 0.02, *p* < .001), peer victimization (*Est.* = 0.20, *SE* = 0.03, *p* < .001), sexual activity (*Est.* = 0.21, *SE* = 0.04, *p* < .001), and attitudes supporting gender inequality (*Est.* = 0.09, *SE* = 0.03, *p* < .01) were all significantly associated with higher verbal TDV victimization. Similarly, among boys, peers' justification of dating violence (*Est.* = 0.04, *SE* = 0.02, *p* < .05), peer victimization (*Est.* = 0.18, *SE* = 0.03, *p* < .001), sexual activity (*Est.* = 0.15, *SE* = 0.04, *p* < .001), and attitudes supporting gender inequality (*Est.* = 0.14, *SE* = 0.03, *p* < .001) were all significantly associated with higher verbal TDV victimization.

**Table 3.** Verbal TDV Victimization for Girls and Boys.

	Girls		Boys	
	Verbal TDV		Verbal TDV	
	Est.	*SE*	Est.	*SE*
Peers' justification of TDV	0.10***	**0.02**	0.04*	**0.02**
Peer victimization	0.20***	**0.03**	0.18***	**0.03**
Sexual activity	0.21***	**0.04**	0.15***	**0.04**
Attitudes supporting gender inequality	0.09**	**0.03**	0.14***	**0.03**
Age	0.03	0.02	0.02	0.01
Black/African American	−0.08	0.05	−0.05	0.04
Hispanic	−0.06	0.05	−0.07	0.04
Other race/ethnicity	0.06	0.06	0.02	0.05

*Note.* TDV = teen dating violence. *SE* = standard error.

*** *p* < .001; ** p < .01; * p < .05.

White was the reference category for each race/ethnicity. Boys was the reference category for gender.

### Physical TDV Victimization by Gender

Table 4 presents results for the physical TDV victimization models for both boys and girls. Among girls, peers' justifications of dating violence (*Est.* = 0.05, *SE* = 0.02, *p* < .01), peer victimization (*Est.* = 0.11, *SE* = 0.03, *p* < .001), and sexual activity (*Est.* = 0.08, *SE* = 0.03, *p* < .01) were significantly associated with higher physical TDV victimization. Among boys, peers' justification of dating violence (*Est.* = 0.04, *SE* = 0.02, *p* < .05), peer victimization (*Est.* = 0.05, *SE* = 0.02, *p* < .01), and attitudes supporting gender inequality (*Est.* = 0.06, *SE* = 0.02, *p* < .01) were significantly associated with higher physical TDV victimization. Lastly, being Hispanic (*Est.* = −0.06, *SE* = 0.02, *p* < .01) was significantly associated with lower physical TDV when compared to being White.

**Table 4.** Physical TDV Victimization for Girls and Boys.

	Girls		Boys	
	Physical TDV		Physical TDV	
	Est.	*SE*	Est.	*SE*
Peers' justification of TDV	0.05**	**0.02**	0.04*	**0.02**
Peer victimization	0.11***	**0.03**	0.05**	**0.02**
Sexual activity	0.08**	**0.03**	0.02	0.03
Attitudes supporting gender inequality	0.02	0.02	0.06**	**0.02**
Age	0.01	0.01	0.01	0.01
Black/African American	0.03	0.02	0.01	0.03
Hispanic	0.00	0.03	−0.06**	**0.02**
Other race/ethnicity	0.08	0.04	0.03	0.04

*Note.* *** *p* < .001; ** p < .01; * p < .05. TDV = teen dating violence. *SE* = standard error.

White was the reference category for each race/ethnicity. Boys was the reference category for gender.

## Discussion

The current study utilized a cross-sectional design to identify how the following attitudes and behaviors were associated with TDV: (1) peers’ justification of TDV, (2) peer victimization, (3) sexual activity, and (4) attitudes supporting gender inequality, as well as age, gender, and race/ethnicity. Given the extant literature that had identified gender differences in TDV victimization, this study examined boys and girls separately to further understand how these associations varied. Many studies have examined associations between social learning in the family context (e.g., domestic violence) and TDV victimization, but the existing literature lacks a deeper understanding of the influence peers may have on TDV victimization. In addition, examining peer victimization can provide valuable insight into prevention efforts for polyvictimization among high school students. Furthermore, sexual activity and attitudes supporting gender inequality have different social repercussions for boys and girls, and thus merit close attention when considering them as risk factors for TDV victimization.

Victimization does not occur in a vacuum, and there are numerous variables that further complicate TDV prevention efforts. While extant research demonstrated the importance of family in the development of belief systems (Sheidow et al., 2001), studies have shown that specifically during adolescence, attitudes and behaviors regarding relationships and societal norms are significantly influenced by peers (Oudekerk et al., 2014). This finding was explored in the current study because research shows that normative beliefs about aggression are among the strongest factors associated with TDV victimization (O’Keefe, 2005). In the current study of high school students, both peers’ justification of TDV and peer victimization were significantly and positively associated with verbal and physical TDV victimization. This finding remained throughout all analyses, including when analyses were conducted separately by gender.

Although each of the factors considered in this study relate to the overarching social learning theoretical framework, these factors are most directly related to social learning and the imitation of behaviors. Having peers who justify dating violence was associated with higher levels of TDV victimization for both adolescent males and females. This finding offers support to previous literature by correlating risky peer networks with TDV (Chase et al., 2002). Similarly, being victimized by peers was associated with being victimized within a romantic relationship. Prevention programs for TDV should continue to address interpersonal violence in the home and incorporate strategies for TDV prevention among peers in school-based or community-based settings. For example, *CDC’s Dating Matters®: Strategies to Promote Healthy Teen Relationships (Dating Matters)* is a comprehensive TDV prevention model for middle school students that incorporates a parent program and a youth program aimed at encouraging peers to promote healthy teen relationships (Estefan et al., 2021). However, this program is limited to middle school students and should be expanded to high schools given our findings that peer influence continues to be an important predictor for TDV victimization among high school students. During adolescence, a sense of belonging among peers becomes increasingly important (Oudekerk et al., 2014). Thus, opportunities for effective prevention and intervention strategies are missed when we do not provide youth-led programming to encourage healthy relationships at the high school level.

In this study, other factors such as sexual activity and gender inequality attitudes were also associated with verbal and physical TDV victimization. Sexual activity was significantly associated with verbal TDV victimization for the overall sample, and physical TDV among girls. This finding is consistent with previous literature, which suggests that having sex before the age of 16 years is correlated with higher levels of TDV victimization (Lichter & McCloskey, 2004; Rodgers & McGuire, 2012). Although our sample ranged from 13-17 years old, the vast majority (93%) of participants were under 16 years old.

Relatedly, attitudes in support of gender inequality or sexist attitudes were significantly associated with verbal TDV victimization for the overall sample. This finding is consistent with previous literature (Reyes et al., 2016). Interestingly, however, attitudes in support of gender inequality were significantly associated with higher rates of physical TDV among boys, but not girls. This is unaligned with previous literature, which suggests that these attitudes increase males’ *perpetration* of dating violence, and females’ risk of *victimization* (McCauley et al., 2013; Reed et al., 2011; Taquette & Monteiro, 2019). This could potentially be explained by the knowledge that perpetrators of violence are often also simultaneously victims of violence, and vice versa (Palmetto et al., 2013). A longitudinal study on perpetration found that traditional gender role attitudes were positively associated with an increased risk for dating violence perpetration 18 months later (Reyes et al., 2016). It is probable that over time, verbal TDV victimization among those who hold sexist views may also increase their chances of physical TDV victimization, but that trajectory may differ among boys and girls (Wincentak et al., 2017). TDV prevention and intervention efforts should also address sexism to dismantle traditional gender roles that can normalize unhealthy or unbalanced relationships that are harmful to both boys and girls. However, given that all tests of moderation by gender were nonsignificant, differing patterns for boys and girls in the separate models should be interpreted with caution.

### Limitations

Despite the strengths of this study, there were several limitations. First, this study was cross-sectional, meaning that it did not analyze behaviors over an extended period of time and cannot explain TDV victimization trajectories across development. As this study did not specify when the dating violence occurred, directionality cannot be suggested, limiting the implications that can be drawn. Similarly, this study did not specify when the *onset* of sexual activity occurred for each participant. Although only 7% (*N* = 132 out of 1884 participants) of participants were ages 16 or 17, this imperfect measure of sexual initiation could slightly skew the implications that are drawn for “risky” sexual behavior, defined previously as sexual initiation before the age of 16 (Lichter & McCloskey, 2004; Rodgers & McGuire, 2012)..

Another limitation is that participants were recruited from a Midwestern U.S. state, which limits the generalizability of the findings to other geographic areas or more diverse samples. Additionally, although gender and sexual minority groups are disproportionately victims of TDV (CDC, 2020), this study was underpowered to examine how participants’ gender identities and sexual orientations were associated with TDV victimization. Similarly, due to the lack of significance of all gender moderation analyses, we recommend caution when interpreting gender differences in the current study. Future studies should take these factors into consideration to reach a deeper understanding of these findings.

### Implications and Future Research

Verbal and physical TDV victimization have profound impacts on the well-being of adolescents. Evidence-based TDV prevention programs including *CDC’s Dating Matters®: Strategies to Promote Healthy Teen Relationships (Dating Matters)* and *Shifting Boundaries* incorporate a comprehensive TDV prevention model for middle school students. Given our sample of high school students, there is a need for evidence-based TDV programs to include older and younger populations. TDV prevention efforts could start as early as elementary school to teach students about healthy interpersonal relationships. Social Emotional Learning (SEL) programming is currently being introduced in schools across the U.S. at varying grade levels to facilitate positive intra- and interpersonal relationships, self-regulation, and responsible decision making while recognizing shifts in peer dynamics during development. This is especially important given that involvement in peer violence peaks in middle school, and both boys and girls who are victimized by peers may be at a higher risk of TDV (Espelage & Holt, 2007).

Additionally, TDV prevention efforts should also consider introducing comprehensive sexual education to encourage healthy romantic relationships and promote safe consensual sex among teens already having sex. Sexual activity, which can be considered an indicator of early sexual debut given the age of this sample, was associated with verbal TDV victimization for both genders and has been associated in the literature with other long-term negative outcomes (e.g., risky sexual behavior and substance use). Extant literature on comprehensive sexual education has found that besides helping delay the onset of sex, it can also reduce risky sexual behaviors (e.g., unprotected sex, teen pregnancy, and sexually transmitted diseases; Stanger-Hall & Hall, 2011; Starkman & Rajani, 2001), as well as the emotional consequences previously mentioned.

Supporting traditional gender norms was significantly associated with verbal victimization for all adolescents and physical victimization for male adolescents, which suggests that sexist attitudes place all adolescents at risk of TDV victimization. Prevention efforts should address sexism along with other predictors of gender-based violence, including heteronormativity and cisnormativity. Future studies should consider how these variables influence TDV victimization and perpetration with longitudinal data and a diverse sample of participants, including sexual and gender minority youth (SGMY).
